# Antioxidant, Enzyme-Inhibitory and Antimicrobial Activity of Underutilized Wheat and Maize Crop Residues

**DOI:** 10.3390/plants14030346

**Published:** 2025-01-24

**Authors:** Stevan Samardžić, Ivona Veličković, Marina T. Milenković, Jelena Arsenijević, Djordje Medarević, Zoran Maksimović

**Affiliations:** 1 Department of Pharmacognosy, University of Belgrade—Faculty of Pharmacy, Vojvode Stepe 450, 11221 Belgrade, Serbia; jelena.arsenijevic@pharmacy.bg.ac.rs (J.A.); zoran.maksimovic@pharmacy.bg.ac.rs (Z.M.); 2 Institute of Botany and Botanical Garden “Jevremovac”, University of Belgrade—Faculty of Biology, 11000 Belgrade, Serbia; ivona@bio.bg.ac.rs; 3 Department of Microbiology and Immunology, University of Belgrade—Faculty of Pharmacy, Vojvode Stepe 450, 11221 Belgrade, Serbia; marina.milenkovic@pharmacy.bg.ac.rs; 4 Department of Pharmaceutical Technology and Cosmetology, University of Belgrade—Faculty of Pharmacy, Vojvode Stepe 450, 11221 Belgrade, Serbia; djordje.medarevic@pharmacy.bg.ac.rs

**Keywords:** *Triticum aestivum* L., *Zea mays* L., tricin, salcolin A, salcolin B, *p*-coumaric acid, ferulic acid, antioxidant activity, enzyme-inhibitory activity, antimicrobial activity

## Abstract

Global wheat and maize production, which reached two billion tonnes in 2021, generates significant agricultural waste with largely untapped potential. This study investigates the bioactive properties of ethanol extracts from wheat and maize harvest residues, their ethyl acetate fractions, and their principal compounds. In vitro assays (DPPH, ABTS, FRAP, and TRC) revealed variable antioxidant capacities among the samples, with ferulic acid demonstrating the strongest free-radical scavenging and reducing effects, often surpassing those of standard antioxidant controls. Enzyme inhibition assays identified the flavonoid tricin as the most effective inhibitor of α-glucosidase, acetylcholinesterase, and butyrylcholinesterase, while the flavonolignan mixture of salcolins A and B showed the highest inhibitory activity against α-amylase and tyrosinase. Antimicrobial testing using the broth microdilution method resulted in minimum inhibitory concentrations (MICs) ranging from 31.25 µg/mL to >1000 µg/mL. Gram-positive bacteria showed the highest susceptibility, *Candida albicans* exhibited variable sensitivity, and Gram-negative bacteria were resistant in the tested concentration range. Bioactivity increased in the order of extracts, fractions, and then individual compounds. These findings suggest that wheat and maize residues possess notable bioactive properties, highlighting their potential as sources of valuable and pharmacologically active compounds.

## 1. Introduction

According to the Food and Agriculture Organization of the United Nations, global primary crop production increased by 54% between 2000 and 2021, reaching 9.5 billion tonnes in 2021. Sugarcane, maize, wheat, and rice accounted for nearly half of this total, with sugarcane leading at 20% (1.9 billion tonnes), followed by maize at 13% (1.2 billion tonnes), and wheat and rice each at 8% (0.8 billion tonnes) [1]. This scale of agricultural production generates substantial amounts of crop residues, such as maize stover and wheat straw, which remain in fields post-harvest. While a small portion of these residues is used for bioethanol production and animal feed, the majority is discarded or burned, contributing to environmental pollution and posing health risks. Given the vast quantities of agricultural waste, various strategies are being developed to enhance agricultural sustainability through improved waste management. These strategies encompass a wide range of applications, including animal feed, roof thatching, surface mulching, composting, fertilizers, direct combustion for energy, fibers for the textile industry, bio-bricks, paper and pulp production, mushroom cultivation, adsorbents for heavy metal removal, organic acid production, industrial enzymes, and biofuel generation [2]. Despite these applications, significant potential remains for better utilization of discarded plant materials.

Recent studies have investigated the potential of wheat and maize field residues in the pharmaceutical, cosmetic, and food industries. These investigations have focused on the production and refinement of microcrystalline cellulose from discarded plant materials, which can serve as an excipient in the production of pharmaceutical solid dosage forms [3]. Additionally, the incorporation of extracts from these residues into meat products and semi-solid skincare formulations has been explored to assess their effects on product properties and consumer acceptance [4,5]. To further explore the bioactivity of wheat and maize harvest residues, the present study evaluated the antioxidant, enzyme-inhibitory, and antimicrobial activities of their ethanol extracts, their phenolic-rich ethyl acetate fractions, as well as their dominant compounds. This approach aimed to estimate the contribution of ethyl acetate fractions and individual compounds to the overall bioactivity of the extracts.

## 2. Results and Discussion

### 2.1. Isolated Compounds

The application of dry column vacuum chromatography, Sephadex LH-20 column chromatography, and thin-layer silica gel separation led to the isolation of compound **1** and a mixture of compounds **2** and **3** from the wheat ethanol extract. These compounds and market-sourced phenolic acids (*p*-coumaric acid and ferulic acid) were used in subsequent bioactivity testing. Compound **1** was identified as the flavonoid tricin (5,7,4′-trihydroxy-3′,5′-dimethoxyflavone) by comparing its UV, MS, and NMR spectra with those of a commercially available reference standard. The mixture of compounds **2** and **3** was identified as structurally related flavonolignans, salcolin A (tricin 4′-*O*-(*threo*-*β*-guaiacylglyceryl)ether) and salcolin B (tricin 4′-*O*-(*erythro*-*β*-guaiacylglyceryl)ether), based on literature data [6,7]. These two compounds were present in a 1:1 ratio, as determined from peak/signal areas in the HPLC-DAD chromatogram and ^1^H NMR spectra. Tricin has previously been reported in wheat hulls and leaves [8,9] and in maize stems [10], while salcolins A and B have been isolated from maize stems [10]. Notably, wheat hull has served as a source for isolating seven flavonolignans structurally similar to salcolins A and B [8]. To our knowledge, this is the first study to report the presence of salcolins A and B in wheat.

The experimentally generated spectral data for the isolated compounds are provided below.

Compound **1** (tricin) (Figure S1, Supplementary Materials). UV λ_max_ 268, 352 nm; ESI-MS *m*/*z* 329.0 [M–H]^−^; ^1^H NMR (400 MHz, CD_3_COCD_3_) δ 13.02 (s, 1H, 5-OH), 7.39 (s, 2H, H-2′,6′), 6.74 (s, 1H, H-3), 6.56 (d, *J* = 2.0 Hz, 1H, H-8), 6.26 (d, *J* = 2.0 Hz, 1H, H-6), 3.97 (s, 6H, 3′5′-OCH_3_).

Mixture of compounds **2** and **3** (1:1) (salcolins A and B) (Figure S2, Supplementary Materials). In the NMR spectra, atoms with the letter “a” correspond to salcolin A, while those with “b” correspond to salcolin B. UV λ_max_ 270, 336 nm; ESI-MS *m*/*z* 525.1 [M–H]^−^; ^1^H NMR (400 MHz, CD_3_COCD_3_) δ 12.88 (s, 2H, 5-OHa, 5-OHb), 7.41 (s, 4H, H-2′,6′a, H-2′,6′b), 7.09–7.05 (m, 2H, H-15a, H-15b), 6.93 (dd, *J* = 8.3, 1.6 Hz, 1H, H-19a), 6.88 (dd, *J* = 8.1, 1.2 Hz, 1H, H-19b), 6.81–6.75 (m, 4H, H-3a, H-3b, H-18a, H-18b), 6.55 (d, *J* = 1.8 Hz, 2H, H-8a, H-8b), 6.26 (d, *J* = 1.8 Hz, 2H, H-6a, H-6b), 5.07–5.01 (m, 2H, H-13a, H-13b), 4.38–4.32 (m, 1H, H-12b), 4.20–4.14 (m, 1H, H-12a), 4.03 (s, 6H, 3′5′-OCH_3_a), 4.01 (s, 6H, 3′5′-OCH_3_b), 3.90 (dd, *J* = 11.6, 4.7 Hz, 1H, 11-CH_2_b), 3.84 (s, 3H, 16-OCH_3_b), 3.83 (s, 3H, 16-OCH_3_a), 3.75 (dd, *J* = 12.2, 3.6 Hz, 1H, 11-CH_2_a), 3.54 (dd, *J* = 11.9, 3.1 Hz, 1H, 11-CH_2_b), 3.39 (dd, *J* = 12.2, 3.3 Hz, 1H, 11-CH_2_a).

^13^C NMR (101 MHz, CD_3_COCD_3_) δ 182.93 (C-4a, C-4b), 166.74 (C-7a, C-7b), 164.09 (C-2b), 164.02 (C-2a), 163.29 (C-5a, C-5b), 158.95 (C-9a, C-9b), 154.66 (C-3′,5′b), 154.35 (C-3′,5′a), 148.03 (C-16a), 147.98 (C-16b), 146.85 (C-17a), 146.59 (C-17b), 140.44 (C-4′a), 140.00 (C-4′b), 133.76 (C-14b), 133.66 (C-14a), 127.88 (C-1′a), 127.80 (C-1′b), 120.67 (C-19a), 120.17 (C-19b), 115.25 (C-18b), 115.24 (C-18a), 111.42 (C-15a), 111.01 (C-15b), 105.98 (C-3a), 105.94 (C-3b), 105.01 (C-10b), 104.96 (C-10a, C-2′,6′b), 104.93 (C-2′,6′a), 100.26 (C-6a, C-6b), 95.27 (C-8a, C-8b), 89.75 (C-12a), 88.15 (C-12b), 73.89 (C-13a), 73.60 (C-13b), 61.65 (C-11a), 61.15 (C-11b), 57.00 (3′,5′-OCH_3_a), 56.97 (3′,5′-OCH_3_b), 56.28 (16-OCH_3_b), 56.25 (16-OCH_3_a).

Assignments of carbon atoms are presented in Figures S1 and S2 (Supplementary Materials).

### 2.2. Chemical Composition of Wheat and Maize Harvest Residues

In this study, the ethanol extracts of wheat and maize harvest residues (WEE and MEE) and their ethyl acetate fractions (WEF and MEF) were subjected to chemical analysis and bioactivity testing. The ethyl acetate fraction yields from the ethanol extracts were 35.32% for wheat and 48.27% for maize. Spectrophotometric analysis showed that both the total phenolic content (TPC) and total flavonoid content (TFC) were higher in WEF (TPC = 27.83 mg/g, TFC = 22.48 mg/g) than in MEF (TPC = 12.41 mg/g, TFC = 7.54 mg/g). The concentration of ferulic acid, determined by LC-DAD-ESI-MS, was greater in WEE than in MEE (1.80 mg/g vs. 1.22 mg/g). Conversely, MEE contained higher levels of salcolins A and B compared to WEE (6.19 mg/g vs. 4.37 mg/g) (Table 1). Additionally, as part of our previous investigations into the potential applications of wheat and maize harvest residues in food and cosmetic products formulation [4,5], we reported data on their chemical composition, which are also presented in Table 1 to facilitate comparisons. These findings show that WEE is richer in total phenolics (23.19 mg/g vs. 20.44 mg/g), total flavonoids (15.22 mg/g vs. 9.37 mg/g), *p*-coumaric acid (2.14 mg/g vs. 1.64 mg/g), and tricin (3.92 mg/g vs. 1.99 mg/g) compared to MEE. A visual comparison of the main constituents and chemical parameters in WEE and MEE is provided in Figure S3 of the Supplementary Materials, highlighting that salcolins A and B constitute the largest share of quantified components in both extracts. The applied separation procedure, i.e., liquid–liquid extraction, differentially affected the concentrations of individual phenolic compounds. HPLC-DAD-ESI-MS profiling showed that tricin and salcolins A and B were concentrated in the ethyl acetate fractions, while *p*-coumaric acid and ferulic acid levels decreased. This outcome aligns with expectations, as the distribution of molecules between immiscible phases (water and ethyl acetate) depends on molecular polarity, among other factors.

Previous studies on various parts of the wheat plant, including unfermented straw, whole grains, and grass, reported TPC values that ranged widely from 2.1 to 210.8 mg/g, while TFC values were reported at 9.9 mg/g and 37.9 mg/g based on two samples analyzed for this parameter [11,12,13,14]. Similarly, TPCs in samples from maize unfermented straw, straw peels, straw core, husk leaf, whole grains, cob, silk, and seeds showed considerable variation, ranging from 0.1 to 215.1 mg/g, while TFCs ranged from 0.1 to 182.3 mg/g [12,13,15,16,17,18,19,20]. Direct comparisons of these with our study results are challenging due to differences in the plant parts studied, extraction solvents, results expression, and other factors; however, literature values generally fall within a low-to-moderate range, aligning with those observed for our samples.

Galletti et al. (1988) analyzed the chemical composition of free phenolics in wheat straw using HPLC and found ferulic acid at a concentration of 8.78 mg per 100 g of straw [21]. Similarly, Alghamdi et al. (2022) examined an aqueous extract of wheat straw, reporting ferulic acid at 103.37 µg/g in the dry extract [22]. Both of these concentrations are lower than that observed in our study. This discrepancy may be due to the fact that Galletti et al. expressed concentration per 100 g of straw, whereas we expressed it per 1 g of extract, resulting in an expectedly higher value per gram of extract. For Alghamdi et al., the difference might be related to the extraction solvent used, as water may be less effective than ethanol in extracting hydroxycinnamic acid derivatives like ferulic acid. Our ferulic acid level was lower than that reported by Đorđević et al. (2019), who used HPLC to analyze methanol extract of unfermented wheat straw and found a concentration of 4.64 mg/g [12]. Although the ferulic acid content in our extract was 2.6 times lower, these levels are still comparable, as both studies reported ferulic acid within a low concentration range (0.1–1%).

While salcolins A and B have been isolated from maize stem [10], no data are available on their levels in maize materials. Similarly, there is no information on the concentrations of salcolins A and B in wheat tissues.

### 2.3. Antioxidant Activity

The ethanol extracts of wheat (WEE) and maize (MEE) harvest residues, along with their corresponding ethyl acetate fractions (WEF and MEF), exhibited notable radical scavenging and reducing activities, although these were generally lower than those of the control substances, butylated hydroxytoluene (BHT) and L-ascorbic acid (Table 2). In the ABTS and TRC assays, WEE demonstrated higher activity than MEE, whereas MEE showed greater potency in the DPPH and FRAP assays. Despite statistically significant differences among the extracts, their activity levels could be considered comparable. The ethyl acetate fractions generally showed higher activity than their respective ethanol extracts, with the exception of WEF in the DPPH assay. MEF exhibited stronger activity than WEF in the DPPH, ABTS, and FRAP assays, while WEF showed superior reducing power in the TRC assay. Similar to the ethanol extracts, the ethyl acetate fractions displayed comparable effects across different tests. Control substances BHT and L-ascorbic acid demonstrated significantly higher activities, except in the case of MEF in the FRAP assay. Notably, reducing capacity of MEF exceeded that of BHT, with a FRAP value of 0.62 µmol Fe^2^⁺/g compared to BHT’s 0.41 µmol Fe^2^⁺/g.

The dominant phenolic compounds in the tested extracts exhibited stronger activities than both the wheat (WEE) and maize ethanol extracts (MEE), as well as their corresponding ethyl acetate fractions, and in some assays, their activity surpassed that of the control substances (Table 3). In the DPPH assay, ferulic acid demonstrated notable scavenging activity (IC_50_ = 6.8 µg/mL), positioning its potency between BHT and L-ascorbic acid. The mixture of salcolins A and B was less potent, while *p*-coumaric acid and tricin exhibited the lowest activities. In the ABTS assay, ferulic acid (IC_50_ = 3.4 µg/mL) was the most effective at neutralizing free radicals, even surpassing the control substances. The IC_50_ values of *p*-coumaric acid and salcolins A and B mixture were also below those of L-ascorbic acid (IC_50_ = 31.6 µg/mL) and BHT (IC_50_ = 40 µg/mL), whereas tricin demonstrated the lowest capacity to neutralize ABTS radicals. In the FRAP assay, which measures reducing properties, ferulic acid exhibited the highest activity (2.02 µmol Fe^2^⁺/g) among all tested compounds. The reducing power of *p*-coumaric acid and the salcolins A and B mixture were intermediate, falling between those of BHT and L-ascorbic acid, while tricin displayed the lowest potency. TRC results further confirmed ferulic acid’s superior antioxidant potential, with the highest activity (2467.90 mg L-AAE/g) among the compounds tested. *p*-Coumaric acid (1810.89 mg L-AAE/g) outperformed BHT, whereas the salcolins A and B mixture, along with tricin, exhibited the weakest activity in the TRC assay.

The in vitro assays identified ferulic acid as the most promising compound in terms of antioxidant potency among those tested, while tricin consistently exhibited the lowest activity, remaining below those of the control substances. However, these results should be interpreted with caution, as they were obtained through in vitro testing, and the compound potency may change in vivo due to factors such as their pharmacokinetic behavior. The lower activity of the extracts and fractions compared to isolated compounds can likely be attributed to the complexity of the extracts and fractions, which contain a mixture of compounds with varying antioxidant capacities. While the data show that the ethanol extracts of harvest residues possess relatively weak antioxidant properties, they may still serve as a valuable source of bioactive compounds with potent antioxidant activity. The ethyl acetate fractions demonstrated better activity than the ethanol extracts, though their effectiveness remained lower than those of the control substances.

As could be expected, much of the prior research has focused on the antioxidant properties of wheat and maize materials used for human consumption or as dietary supplements, such as wheat and maize grains or wheatgrass juice. In contrast, crop residues left in the field after harvest have generally been regarded as low-value material and, as such, have received less scientific attention.

Đorđević et al. (2019) investigated the antioxidant activity of methanol extracts from unfermented wheat and maize straw using the DPPH assay [12]. They determined that the IC_50_ value for the wheat extract was 0.55 mg/mL, while the maize extract exhibited more than twice the potency (IC_50_ = 0.23 mg/mL). In comparison, the control substance BHT (IC_50_ = 0.05 mg/mL) showed stronger activity than both extracts. These findings align well with our results, where wheat extract also demonstrated weaker antioxidant activity compared to maize extract. Furthermore, Đorđević et al. showed that in the FRAP assay, the wheat methanol extract (573.3 µmol Fe(II)/g DE) exhibited greater reducing power than the maize methanol extract (442.2 µmol Fe(II)/g DE). Although our study revealed a lower FRAP value for the wheat ethanol extract, this discrepancy is likely attributable to differences in plant material origin, extraction solvents, and experimental conditions.

Additional support for our findings comes from the recent work by Yang et al. (2024), who evaluated the ability of ethyl acetate fractions from hydroethanolic extracts of defatted maize silk, straw peels, and straw core to neutralize free radicals [20]. Their IC_50_ values in the DPPH assay ranged from 0.24 to 0.61 mg/mL (BHT IC_50_ = 0.15 mg/mL), which are comparable to the values observed in our study. However, in the ABTS assay, maize samples exhibited stronger activity (IC_50_ = 0.05–0.13 mg/mL) than BHT (IC_50_ = 0.14 mg/mL), a result opposite to what we observed, where the control substances were the more potent agents.

When Sahin et al. (2017) assessed the DPPH activity of methanol extracts from de-hulled grains of five *T. aestivum* cultivars at concentrations ranging from 12.5 to 200 µg/mL, they found DPPH inhibition to range from 0.15% to 16.11% [23]. By comparison, ascorbic acid applied at the same concentrations achieved 96.29% to 96.86% inhibition. Studies on the antioxidant activity of wheat sprout juice and seeds in the DPPH assay reported a wide range of IC_50_ values (103–14400 µg/mL) [24,25]. In the case of maize, water extracts of maize silk had an IC_50_ of 0.68 mg/mL in the DPPH assay [18], while hydroethanolic extract yielded an IC_50_ of 116.2 µg/mL [19]. Moreover, Dewi et al. (2021) investigated the hydroethanolic extract of maize cobs and reported its ability to reduce DPPH radical concentration with an IC_50_ of 38.57 µg/mL, which was less potent than ascorbic acid (IC_50_ = 3.55 µg/mL) [15]. In the study by Elemosho et al. (2021), methanol extracts of flours from six yellow-orange maize hybrids exhibited IC_50_ values ranging from 9.07 mg/mL to 26.35 mg/mL in the DPPH assay. These values were higher than the IC_50_ value of the control substance, ascorbic acid (4.63 mg/mL) [26]. Similarly, an investigation of ethanol extracts from maize tassels demonstrated limited radical-scavenging activity. At a concentration of 0.2 mg/mL, the extract scavenged 7.04% of DPPH radicals and 9.45% of ABTS radicals, whereas butylated hydroxytoluene at the same concentration achieved significantly higher inhibition, 47.32% and 50.32%, respectively [27]. Furthermore, Barnawi et al. (2023) reported the antioxidant capacity of methanol extracts from maize pollen grains, which exhibited an IC_50_ value of 54.85 µg/mL in the DPPH assay. However, the IC_50_ value for ascorbic acid in the same study was markedly lower (2.51 µg/mL), highlighting its superior antioxidant efficacy [28]. This wide variability in IC_50_ values across studies on wheat de-hulled grains, sprout juice, and seeds, as well as maize silk, cobs, flour, tassel, and pollen, makes direct comparisons with our findings challenging.

Jeong et al. (2010) demonstrated that *p*-coumaric acid and ferulic acid, tested at concentrations of 50–1000 µg/mL, inhibited DPPH radicals by 10.3–26.9% and 84.3–92.3%, respectively [24]. BHT, tested at the same concentration range, displayed 73.9–90.7% inhibition, making it a weaker antioxidant than ferulic acid. These results are consistent with our findings, where ferulic acid also showed greater antioxidant potency compared to *p*-coumaric acid and BHT. Mohanlal et al. (2011) examined the DPPH inhibitory properties of tricin and flavonolignans, salcolins A and B, isolated from medicinal rice (*Oryza sativa* L.) [7]. They reported IC_50_ values of 90.39 µg/mL for tricin, 208.1 µg/mL for salcolin A, and 352.04 µg/mL for salcolin B. The IC_50_ of the control substance quercetin was 42.98 µg/mL. Our findings align with these results for salcolins A and B, though in our study, tricin exhibited lower activity than the salcolins.

### 2.4. Enzyme-Inhibitory Activity

The ethanol extracts of wheat and maize field residues were tested in vitro against five enzymes relevant to the treatment of diabetes, Alzheimer’s disease, and skin conditions. These experiments aimed to explore the potential of underutilized agricultural by-products as raw materials for the pharmaceutical and cosmetic industries. In this way, value could be added to widely available yet currently underexploited resources. Their activities (IC_50_ = 5.80–>100 mg/mL) were consistently lower than those of the control substances (IC_50_ = 0.34–9.87 mg/mL), some of which are clinically used drugs (Table 4). In general, the ethyl acetate fractions exhibited greater potency, suggesting that the active constituents were concentrated by a simple liquid–liquid extraction procedure in a separatory funnel. WEE displayed stronger activity than MEE in the *α*-amylase and BChE assays, while MEE was more potent against *α*-glucosidase, AChE, and tyrosinase. Although the IC_50_ values for WEE and MEE differed significantly, the overall activity levels were comparable. MEF showed lower IC_50_ values than WEF in most assays, except for AChE, where WEF demonstrated stronger activity. In terms of potency (IC_50_ = 0.11–59.31 mg/mL), the ethyl acetate fractions generally outperformed the ethanol extracts, though they were less potent than the control substances. However, MEF was more effective against *α*-glucosidase (IC_50_ = 0.11 mg/mL) than acarbose (IC_50_ = 0.37 mg/mL), highlighting its potential.

As expected, the tested compounds generally showed greater enzyme inhibitory potency than the ethanol extracts and their ethyl acetate fractions. However, some compounds lacked inhibitory effects within the tested concentration range. Their IC_50_ values spanned from 0.19 mg/mL to 27.44 mg/mL, but in the tyrosinase inhibition assay, due to low activity of most compounds, IC_50_ could only be determined for the salcolins A and B mixture (Table 5). In the *α*-amylase inhibition test, acarbose (IC_50_ = 0.43 mg/mL) was the most effective inhibitor, followed by the salcolins A and B mixture, tricin, and ferulic acid (IC_50_ = 1.73–3.35 mg/mL), with *p*-coumaric acid showing the lowest activity (IC_50_ = 27.44 mg/mL). Tricin was the strongest *α*-glucosidase inhibitor (IC_50_ = 0.19 mg/mL), exceeding the efficacy of the control. Hydroxycinnamic acids and the salcolins A and B mixture displayed modest IC_50_ values, although higher than that of acarbose. All compounds inhibited AChE, with tricin (IC_50_ = 2.88 mg/mL) proving more effective than galantamine (IC_50_ = 9.66 mg/mL), followed by ferulic acid and the salcolins A and B mixture, while *p*-coumaric acid showed the lowest activity. A similar pattern was observed in the BChE assay, where tricin (IC_50_ = 0.55 mg/mL) was the most potent, surpassing galantamine (IC_50_ = 9.87 mg/mL), with the salcolins A and B mixture and ferulic acid also demonstrating strong activity. Only the salcolins A and B mixture inhibited tyrosinase in the tested concentration range, though its potency was lower than that of the control, kojic acid.

Inhibitory effects varied by enzyme and compound, with some constituents showing strong inhibition of *α*-glucosidase, AChE, and BChE, while others had modest or no effect, as observed in the tyrosinase inhibition assay. These results highlight the potential of wheat and maize harvest residues as sources of pharmaceutically valuable compounds. However, harnessing this potential presents challenges due to the relatively low concentrations of bioactive compounds and the lack of simple isolation methods, underscoring the need for further research into efficient utilization of field residues for such applications.

Previous studies have reported the enzyme-inhibitory properties of various wheat and maize parts in vitro. However, findings are often inconsistent, likely due to differences in the plant parts, extraction solvents, and experimental conditions, making direct comparisons challenging. Nevertheless, these data still hold certain comparative value, as they relate to the same plant species.

Specifically, the methanol extract of wheat sprout juice and its ethyl acetate fraction, at 1.5 mg/mL, inhibited *α*-amylase by 30.4% and 28.6%, respectively, compared to the positive control, acarbose, which achieved 59.3% inhibition. In the *α*-glucosidase assay, the methanol extract demonstrated higher activity (58.2%) than the ethyl acetate fraction (36.1%), while acarbose remained the most effective at 70.2% inhibition [29]. Similarly, methanol extracts of wheat bran and germ at 1 mg/mL inhibited over 80% of *α*-glucosidase activity, although this inhibition dropped below 30% at 0.2 mg/mL [30]. In our study, WEE and WEF also reduced *α*-amylase and *α*-glucosidase activity, although the investigated samples differ from those in previous studies, which examined other wheat parts. Notably, the IC_50_ values in our study were generally higher than those in the literature; however, the observed trend was similar, with both our samples and the previously tested wheat parts demonstrating lower potency than acarbose as the control.

Maize silk has been extensively studied for its enzyme-inhibitory properties. Its hydroethanolic extract exhibited an IC_50_ value of 218.4 µg/mL for *α*-amylase inhibition, with a similar IC_50_ of 221.4 µg/mL for *α*-glucosidase, whereas acarbose showed greater potency with lower IC_50_ values of 105.5 µg/mL and 75.5 µg/mL, respectively [19]. Sabiu et al. (2016) also reported enzyme inhibition by maize silk aqueous extract, with IC_50_ values of 5.89 mg/mL for *α*-amylase and 0.93 mg/mL for *α*-glucosidase, compared to acarbose’s lower IC_50_ values of 3.77 mg/mL and 0.48 mg/mL, respectively [18]. Additionally, Abirami et al. (2021) found that ethanol extract of maize silk inhibited *α*-amylase by 10–67% at concentrations of 0.25–2 mg/mL [31]. Similarly, Mihali et al. (2024) demonstrated that ethanol extracts of maize silk inhibited *α*-glucosidase with an IC_50_ value of 401.67 µg/mL, which was considerably less potent than the standard enzyme inhibitor, acarbose, with an IC_50_ of 52.04 µg/mL [32]. Ethanol extracts from sixteen maize kernel strains inhibited *α*-glucosidase by less than 10% to around 50% at 0.67 mg/mL, depending on the strain, while acarbose inhibited *α*-glucosidase by 62% at 0.44 mg/mL [33]. Elemosho et al. (2021) investigated the enzyme-inhibitory activity of methanol extracts derived from flours of six yellow-orange maize hybrids. The extracts inhibited *α*-amylase and *α*-glucosidase with IC_50_ values ranging from 26.28 to 52.55 mg/mL and 47.72 to 63.98 mg/mL, respectively, whereas acarbose displayed lower IC_50_ values of 24.45 mg/mL for *α*-amylase and 32.88 mg/mL for *α*-glucosidase, confirming its greater inhibitory potency [26]. These studies support the inhibitory activities observed for our maize samples (MEE, MEF); however, direct comparisons are challenging because prior research has primarily focused on maize silk, while our study investigated maize stalks. The observed differences in potency may be attributed to the variation in chemical composition between maize silk and stalks, with maize silk potentially containing higher levels of enzyme-inhibitory compounds.

Studies have also investigated the enzyme-inhibitory activity of isolated compounds. Jeong et al. (2012) found that *p*-coumaric acid exhibited IC_50_ values above 30 mM (4.92 mg/mL) for both *α*-amylase and *α*-glucosidase, while ferulic acid displayed stronger inhibition, with IC_50_ values of 9.5 mM (1.84 mg/mL) and 4.9 mM (0.95 mg/mL), respectively [29]. In comparison, acarbose showed IC_50_ values of 2.4 mM (1.55 mg/mL) and 1.7 mM (1.1 mg/mL) in the same assays. Interestingly, Nile and Park (2014) reported that *p*-coumaric acid (IC_50_ = 90.8 µg/mL) was more effective than acarbose in inhibiting *α*-glucosidase, while ferulic acid (IC_50_ = 251.4 µg/mL) was less effective, with acarbose having an IC_50_ of 230.4 µg/mL in that study [34]. Our findings indicated that both *p*-coumaric and ferulic acid were less potent inhibitors of *α*-amylase and *α*-glucosidase compared to acarbose. Additionally, Wu and Tian (2019) examined tricetin, a compound structurally similar to tricin, and found it to be a more effective inhibitor of *α*-glucosidase than acarbose, though less effective against *α*-amylase [35]. This trend aligns closely with our results for tricin.

The hydroethanolic extract of maize cob demonstrated tyrosinase inhibition, with an IC_50_ of 919.78 µg/mL, while kojic acid, used as a positive control, showed higher potency (IC_50_ = 150.79 µg/mL) [15]. Our results also confirmed the tyrosinase-inhibitory activity of MEE, though with lower potency. In the study by Yucharoen et al. (2023), ethanol extracts of maize silk exhibited significant tyrosinase-inhibitory activity, with an IC_50_ value of 12.45 µg/mL. However, the control substance kojic acid demonstrated greater potency, with an IC_50_ of 4.85 µg/mL [36]. In contrast, Mihali et al. (2024) found that maize silk ethanol extracts, within the tested concentration range, did not exhibit tyrosinase-inhibitory activity [32]. Wille and Berhow (2011) identified 4-hydroxy-1-oxindole-3-acetic acid (tasselin A) from maize tassels and demonstrated its tyrosinase-inhibitory properties, with an IC_50_ value of 0.75 mM [37]. Tricin’s tyrosinase inhibition has been reported with an IC_50_ of 269.46 µg/mL [38]; however, in our study, tricin did not exhibit inhibitory activity within the tested concentration range.

Sim et al. (2014) investigated four *N*-acyl derivatives—tyramine, tryptamine, or serotonin coupled with *p*-coumaric or ferulic acids—isolated from maize stem for their acetylcholinesterase (AChE) inhibitory effects [39]. These compounds showed IC_50_ values ranging from 16.3 to 59.1 µg/mL, while the reference compound berberine exhibited significantly higher potency, with an IC_50_ of 1.5 µg/mL. Such compounds may have contributed to the observed anti-AChE activity in our extract. Additionally, our results pointed to a contribution of dominant compounds in the extracts to AChE inhibition. Kuppusamy et al. (2017) evaluated the AChE inhibitory activity of tricetin, a flavonoid structurally similar to tricin, and found it to be more active (IC_50_ = 18.28 µg/mL) than the clinically used drug donepezil (IC_50_ = 22.00 µg/mL) [40]. This aligns with the low IC_50_ value we observed for tricin. Mihali et al. (2024) reported that maize silk ethanol extract exhibited AChE-inhibitory activity, with an IC_50_ value of 1222.67 µg/mL, which was significantly weaker than the control substance galantamine (IC_50_ = 2.31 µg/mL) [32]. Similarly, Al-Khayri et al. (2022) demonstrated that ethanol extracts of maize tassel inhibited AChE, achieving an IC_50_ value of 0.10 mg/mL; however, no reference compound was included in their study [27]. Direct comparisons between these findings and our results are challenging due to differences in the plant parts examined and variations in experimental methodologies.

### 2.5. Antimicrobial Activity

The antimicrobial activity of ethanol extracts from wheat and maize harvest residues, as well as their individual constituents, was evaluated using the broth microdilution method against eight standard microbial strains: three Gram-positive bacteria, four Gram-negative bacteria, and one yeast strain, *Candida albicans* (Table 6). The tested samples inhibited the growth of Gram-positive bacteria (*Staphylococcus aureus*, *Enterococcus faecalis*, and *Bacillus subtilis*) with minimum inhibitory concentration (MIC) values ranging from 31.25 µg/mL to 500 µg/mL. *B. subtilis* and *E. faecalis* exhibited the highest sensitivity, with MIC values of 31.25 µg/mL for *p*-coumaric acid, the salcolins A and B mixture, and tricin. Of the constituents tested, ferulic acid showed the lowest activity against Gram-positive bacteria, with a MIC of 125 µg/mL against *E. faecalis*. The extracts demonstrated comparable or lower antimicrobial activity compared to the individual constituents. Both wheat ethanol extract (WEE) and maize ethanol extract (MEE) showed equal potency against *B. subtilis* (MIC = 125 µg/mL), but MEE exhibited higher activity against *S. aureus* and *E. faecalis*. In contrast, all tested samples were ineffective against the Gram-negative bacteria, with MIC values exceeding 500 µg/mL or 1000 µg/mL. For *C. albicans*, *p*-coumaric acid and tricin exhibited the highest antifungal activity (MIC = 250 µg/mL), while WEE demonstrated moderate activity (MIC = 500 µg/mL). No significant antifungal effects were observed for the other tested samples at the concentrations used.

These findings suggest that the antimicrobial activity of the extracts is not attributable to a single compound but likely results from the combined effects of multiple compounds. Overall, the results indicate a low to modest antimicrobial activity of the tested extracts and compounds, particularly against Gram-positive bacteria and *C. albicans*, while Gram-negative bacteria were largely resistant.

Glišić et al. (2023) investigated the antimicrobial activity of ethanol extracts from wheat and maize against foodborne pathogens, specifically *Salmonella typhimurium* ATCC 14028, *Salmonella enteritidis* ATCC 13076, *Staphylococcus aureus* ATCC 25923, *Escherichia coli* ATCC 25922, *Listeria monocytogenes* ATCC 19111, and *Yersinia enterocolitica* ATCC 9610 [41]. Reported minimum inhibitory concentration (MIC) values were 320 µg/mL for all tested strains, except for the wheat ethanol extract against *L. monocytogenes*, which exhibited a higher MIC of 640 µg/mL. Lukić et al. (2024) also investigated these extracts, examining their effects on seven bacterial species (both Gram-positive and Gram-negative) and one yeast strain, *Candida albicans* ATCC 24433 [5]. In this study, MIC values were 32 mg/mL and 64 mg/mL across most strains, with the exceptions of maize extract (MIC = 5 mg/mL) and wheat extract (MIC = 1 mg/mL) against *C. albicans*, indicating stronger activity against this yeast. The findings of Glišić et al. suggested similar and relatively high sensitivities of both Gram-positive and Gram-negative bacteria to the wheat and maize extracts, as reflected by their low MIC values. In contrast, Lukić et al. observed that these bacterial groups exhibited similar but relatively low sensitivity, indicated by high MIC values. Our study, however, revealed a significant difference in the responses between Gram-positive and Gram-negative bacteria. For Gram-positive bacteria, our MIC values aligned closely with those of Glišić et al., suggesting comparable levels of activity. For Gram-negative bacteria, our findings were consistent with those of Lukić et al., both showing high MIC values, reflecting lower antimicrobial activity. The observed variations across studies may be due to differences in the bacterial strains used. Glišić et al. focused on foodborne pathogens, while our study targeted human pathogens. Given that even strains within the same bacterial species can exhibit markedly distinct characteristics, these strain differences likely account for the observed discrepancies.

Previous studies have also assessed the antimicrobial potential of wheat bran, wheatgrass, and wheat sprout juice using the agar disk diffusion method [14,24,42]. Tested microorganisms included Gram-positive bacteria (*Lactobacillus rhamnosus*, *Actinomyces viscosus*, *Staphylococcus aureus*, *Sarcina lutea*, *Bacillus pumilus*, *B. subtilis*, *B. cereus*, *Streptococcus salivarius*, *S. mutans*, and *S. sobrinus*), Gram-negative bacteria (*Escherichia coli*, *Bordetella bronchiseptica*, and *Pseudomonas aeruginosa*), and two fungal strains (*Aspergillus fumigatus* and *A. niger*). The effectiveness of the extracts varied widely, depending on the microbial species, type of extract, and specific wheat tissue analyzed, with no clear patterns of antimicrobial activity. Direct comparison of our results with those of previous studies is challenging due to differences in the plant parts and microbial strains used, extraction methods, experimental procedures, and result expression. Nevertheless, our findings generally align with the literature. While our samples primarily inhibited the growth of Gram-positive bacteria, higher extract concentrations may extend their efficacy to Gram-negative bacteria as well.

Maize husk leaf extracts, obtained through successive extractions with solvents of increasing polarity, have been shown to inhibit the growth of *Escherichia coli* (MIC = 175–415 µg/mL), *Bacillus subtilis* (MIC = 180–435 µg/mL), *Aspergillus niger* (MIC = 200–365 µg/mL), and *Fusarium solani* (MIC = 215–415 µg/mL) [17]. In our study, the maize extract displayed a more pronounced activity against *B. subtilis*, though still comparable to that reported by Riaz et al. However, within our tested concentration range, the maize extract did not inhibit the growth of *E. coli* or *Candida albicans*. Studies on other maize parts have also demonstrated antimicrobial potential. Al-Khayri et al. (2022) reported that an ethanol extract of maize tassels inhibited the growth of *Escherichia coli* and *Salmonella typhimurium* (MIC = 312 µg/mL), while *Staphylococcus aureus* growth was unaffected within the tested concentration range (9.75–312 µg/mL) [27]. Yucharoen et al. (2023) found that ethanol extract of maize silk exhibited antibacterial activity against the skin pathogens *Cutibacterium acnes* (MIC = MBC = 15.625 mg/mL) and *Staphylococcus epidermidis* (MIC = MBC = 125 mg/mL) using the broth dilution method [36]. Bae et al. (2021) demonstrated the antimicrobial activity of methanol extracts of maize germ flour, a by-product of the oil industry, against several microbial strains, including *Staphylococcus aureus* (zones of inhibition: 6.28 mm), *Enterococcus hirae* (2.67 mm), *Enterococcus faecalis* (5.77 mm), *Escherichia coli* (6.15 mm), *Pseudomonas aeruginosa* (5.09 mm), *Vibrio harveyi* (5.30 mm), and *Candida albicans* (6.58 mm), with the exception of *Bacillus subtilis*, whose growth was unaffected [43]. Barnawi et al. (2023) investigated the antimicrobial properties of methanol extract of maize pollen grains, reporting MIC values of 3.9 µg/mL for *Bacillus subtilis*, 125 µg/mL for *Staphylococcus aureus*, 250 µg/mL for *Escherichia coli*, 31.25 µg/mL for *Salmonella typhi*, and 15.62 µg/mL for *Candida albicans*, while *Aspergillus niger* remained unaffected [28]. Although these studies highlight the antimicrobial potential of various maize parts, comparison of their findings to ours should be made cautiously due to significant differences in the plant materials tested, experimental designs, and employed microbial strains.

The observed antimicrobial activity of *p*-coumaric acid and ferulic acid in this study is consistent with findings by Jeong et al. (2010), who investigated their effects on oral bacteria [24]. They reported that *p*-coumaric acid inhibited the growth of *Actinomyces viscosus* and *Streptococcus salivarius*, while ferulic acid also suppressed *Streptococcus mutans* in addition to these strains. However, neither phenolic acid showed activity against *Lactobacillus rhamnosus* or *Streptococcus sobrinus* within the tested concentration range. Insights into the antibacterial mechanism of *p*-coumaric acid were provided by Lou et al. (2012), who demonstrated its effectiveness against *Shigella dysenteriae* by disrupting bacterial cell membranes and binding to bacterial genomic DNA [44]. Lou et al. (2012) reported minimum inhibitory concentration (MIC) values of 20 µg/mL for *p*-coumaric acid against three tested Gram-positive bacteria, which align with our findings. However, discrepancies exist in its activity against Gram-negative bacteria, with Lou et al. (2012) reporting MIC values of 10–80 µg/mL, which are lower than those observed in our study. In contrast, Alves et al. (2013) found higher MIC values (≥1 mg/mL) for *p*-coumaric acid and ferulic acid against clinical isolates of both Gram-positive and Gram-negative bacteria, except for ferulic acid’s MIC of 0.5 mg/mL against methicillin-resistant *Staphylococcus aureus* (MRSA) [45]. This variation across studies highlights the potential influence of bacterial strain specificity and experimental conditions on the efficacy of phenolic acids, which may contribute to the observed differences in MIC values.

The antimicrobial activity of tricin and salcolins A and B in our experiment aligns with the recognized antimicrobial properties of many flavonoids. The scientific literature describes several mechanisms by which flavonoids exert these effects, including disruption of bacterial cell membranes, inhibition of cell envelope synthesis, suppression of nucleic acid synthesis, and interference with the electron transport chain and ATP synthesis [46]. Therefore, it is plausible that the activity of these compounds against Gram-positive bacteria is, at least partially, mediated by one or more of these mechanisms.

## 3. Materials and Methods

### 3.1. Materials and Chemicals

Heptane (pro-analysis) and silica gel on TLC Al foils were purchased from Sigma-Aldrich Chemie GmbH (Steinheim, Germany). Methanol (HPLC-grade), ethanol (HPLC-grade), dimethyl sulfoxide (pro-analysis), sodium carbonate anhydrous, and di-sodium hydrogen phosphate dodecahydrate were bought from VWR Chemicals (Lutterworth, UK). Concentrated hydrochloric acid, glacial acetic acid, and ethanol 96% *v/v*(Ph. Eur.) were procured from Zorka Pharma (Šabac, Serbia) and sodium acetate trihydrate from Centrohem (Stara Pazova, Serbia). Quercetin hydrate, 2,2′-azino-bis(3-ethylbenzothiazoline)-6-sulphonic acid (ABTS), and butylated hydroxytoluene (BHT) were obtained from TCI Europe N.V. (Zwijndrecht, Belgium). Merck KGaA (Darmstadt, Germany) provided 2,2-diphenyl-1-picrylhydrazyl (DPPH), *α*-glucosidase from bakers’ yeast (EC 3.2.1.20, type I), *α*-amylase from porcine pancreas (EC 3.2.1.1, type VI-B), acetylcholinesterase (AChE) from *Electrophorus electricus* (EC 3.1.1.7, type VI-S), butyrylcholinesterase (BChE) from equine serum (EC 3.1.1.8), tyrosinase from mushroom (EC 1.14.18.1), *p*-nitrophenyl-*α*-D-glucopyranoside, 5,5′-dithio-bis(2-nitrobenzoic acid) (DTNB), acetylthiocholine iodide (ATCI), butyrylthiocholine iodide (BTCI), 3,4-dihydroxy-L-phenylalanine (L-DOPA), acarbose, galanthamine hydrobromide, kojic acid, iron (II) sulphate heptahydrate, iron (III) chloride hexahydrate, potassium hexacyanoferrate (III), Lugol solution, Folin–Ciocalteu reagent, gallic acid, and silica gel 60 (0.015–0.040 mm) for column chromatography. Thermo Fisher Scientific (Fair Lawn, NJ, USA) supplied potassium peroxydisulphate, trichloroacetic acid, acetonitrile (HPLC-MS grade), hexane (pro-analysis), methanol (pro-analysis), ethyl acetate (pro-analysis), and sodium sulphate anhydrous (pro-analysis). Celite^®^ 545 and 1% starch solution were acquired from Carl Roth GmbH (Karlsruhe, Germany) and Sephadex^®^ LH-20 from Cytiva (Marlborough, MA, USA). 2,4,6-Tris(2-pyridyl)-*s*-triazine (TPTZ) and L-ascorbic acid were sourced from Fluka Chemie AG (Buchs, Switzerland), while formic acid (LC-MS grade) was from Honeywell, Fluka (Seelze, Germany). Tricin was from PhytoLab GmbH & Co. KG (Vestenbergsgreuth, Germany), ferulic acid from Extrasynthese (Genay Cedex, France), and *p*-coumaric acid and acetone-*d*_6_ (99.9% atom D, 0.03% *v*/*v* TMS) from Sigma-Aldrich (St. Louis, MO, USA). Sodium-chloride anhydrous was obtained from Superlab (Belgrade, Serbia).

### 3.2. Plant Material

Plant material collection and extraction were conducted as described by Glišić et al. (2023) [41]. Briefly, harvest residues (wheat straw and maize stalks) were collected from fields in the autonomous province of Vojvodina, Serbia, during July and October, respectively. The collected plant material (3 kg each) was air-dried, milled, and successively extracted in a 60 L industrial stainless-steel extractor. The material was first extracted with hexane at 40 °C for 1 h, followed by drying and a second extraction with 96% (*v*/*v*) ethanol at 45 °C for 1 h. Ethanol was selected as the extraction solvent for several reasons, including its relatively low toxicity, low boiling point, which facilitates the preparation of dry extracts, and its high efficiency in extracting polyphenolic compounds. A plant-to-solvent ratio of 1:6 (m/V) was maintained during each extraction step. The extracts were filtered, and solvents were removed using a rotary vacuum evaporator. The final dry extracts were used for chemical analysis and biological activity assays. Additionally, the ethanol extract of wheat was utilized for isolating dominant secondary metabolites. The final ethanol extracts exhibited a jam-like (semi-solid) consistency, brown color, and pleasant aroma. The ethanol extract yield was 1.35% for wheat and 1.32% for maize.

As liquid–liquid extraction with ethyl acetate may effectively concentrate medium to low polarity polyphenols; we prepared and tested ethyl acetate fractions of the investigated extracts to assess the impact of this simple separation procedure on bioactivity. For fraction preparation, 4 g of the ethanol extract was suspended in 600 mL of water and mixed with four portions of ethyl acetate (4 × 300 mL) in a separatory funnel. The ethyl acetate layers were collected, combined, and filtered over anhydrous sodium sulfate, then evaporated to dryness. These fractions, along with the ethanol extracts, were used in subsequent tests.

### 3.3. Isolation of Dominant Compounds from Wheat Harvest Residues

Wheat ethanol extract was selected as the starting material for isolation. Before separation using dry column vacuum chromatography [47], 2 g of the wheat ethanol extract was dissolved in a minimal volume of ethanol and adsorbed onto Celite using a rotary vacuum evaporator. The adsorbed material was applied to a silica gel column (height = 5.5 cm, diameter = 4 cm, and particle size = 15–40 μm). Elution began with pure heptane, followed by heptane and ethyl acetate mixtures (6:4, 5:5, 4:6, 3:7, 2:8, and 1:9), pure ethyl acetate, and concluded with ethyl acetate and methanol mixtures (9:1, 8:2, 7:3, 6:4, and 5:5). Thirteen fractions (D1–D13, 40 mL each) were collected, and their composition was analyzed by silica gel TLC using a 3:7 *v/v* heptane and ethyl acetate solvent system. Fractions D6–D9, enriched in compound **1**, were pooled and evaporated to dryness. Fractions D10 and D11, containing high levels of compounds **1**, **2**, and **3**, were also combined and evaporated to dryness. The combined D10–D11 fractions were further chromatographed on a Sephadex LH-20 column (height = 33 cm, radius = 1 cm) using 96% ethanol as the eluent, yielding 15 fractions (S1–S15, 20 mL each). Fractions S7 (dominated by compounds **2** and **3**), S8 (dominated by compounds **1**, **2**, and **3**), and S9 and S10 (dominated by compound **1**) were selected for further work. Final purification of fractions D6–D9, S7, S8, and S9–S10 was carried out using TLC (stationary phase: silica gel; mobile phase: heptane/ethyl acetate, 30:70 *v*/*v*). Visualization was performed under UV light at 254 and 365 nm. The zone (R_f_ = 0.60), which quenched fluorescence under both wavelengths, was scraped off, extracted with ethyl acetate, and evaporated to yield compound **1** as a yellow amorphous substance (4.5 mg). Compounds **2** and **3**, appearing as a single zone (R_f_ = 0.26) and also quenching fluorescence under both wavelengths, were isolated in the form of a yellow amorphous solid (5.6 mg), using the same extraction procedure. HPLC analysis confirmed the purity of compound **1** and the mixture of compounds **2** and **3** to be over 95%. To obtain sufficient quantities for bioactivity testing, the chromatographic procedure was repeated several times. Structural characterization of the isolated compounds was based on their UV spectra (190–640 nm) and MS spectra (negative ion mode; fragmentor voltage = 100 V; *m*/*z* range = 100–1000), which was recorded using LC-DAD-ESI-MS. Additionally, ^1^H and ^13^C NMR spectra were acquired on a Bruker Ascend^TM^ 400 instrument at 400 MHz and 101 MHz, respectively, with TMS as the internal standard. Chemical shifts are reported in ppm, with coupling constants in Hz.

### 3.4. Total Phenolic and Total Flavonoid Content Determination

The total phenolic content (TPC) in wheat ethyl acetate fraction (WEF) and maize ethyl acetate fraction (MEF) was determined using the Folin–Ciocalteu reagent, following the method described by Ahmad et al. (2017) [48]. In the same samples, total flavonoid content (TFC) was assessed using the AlCl_3_ microplate reader protocol suggested by Chatatikun and Chiabchalard (2013) [49], with modifications according to Sembiring et al. (2018) [50]. Results were expressed as mg of gallic acid equivalents (GAE) or quercetin equivalents (QE) per gram of dry fraction, respectively.

### 3.5. LC-DAD-ESI-MS Analysis

Ethanol extracts of the wheat and maize harvest residues were examined using an Agilent LC-MS System 1260/6130 (Agilent Technologies, Waldbronn, Germany), which includes a quaternary pump, a column compartment, a DAD detector, an ESI ion source, and a single quadrupole mass detector, following a previously described method [4]. For quantification, ethanol solutions of the extracts were prepared at concentrations of 10 mg/mL and filtered through 0.22 µm membrane filters (Macherey-Nagel, Düren, Germany). Injection volume was 5 µL. Separation was achieved on a reversed-phase Zorbax SB-Aq column (3 × 150 mm, particle size 3.5 µm, Agilent Technologies) maintained at 25 °C. The mobile phase consisted of Solution A (0.1% HOOH) and Solution B (MeCN), with the gradient as follows: 10–90% B (0–25 min), 90% B (25–27 min), and 90–10% B (27–30 min). The flow rate was set at 0.35 mL/min. Chromatograms were recorded at 210, 270, 320, and 350 nm, and UV spectra were recorded over the range 190–640 nm. Mass spectra were acquired over an *m*/*z* range of 100–1000 in negative ion mode with a fragmentor voltage of 100 V. The spray chamber parameters were as follows: gas temperature of 350 °C, drying gas flow of 10 L/min, nebulizer pressure of 40 psig, and capillary voltage of 3500 V. Identification was conducted by comparing retention times, UV, and MS spectra of compounds in the tested extracts with those of the commercially available standard (ferulic acid) and isolated compounds (a mixture of salcolins A and B) under the same chromatographic conditions. Quantification was performed using the external standard method. Calibration curves were established for tricin at 350 nm (y = 43,243.30x + 51.60, R^2^ = 1, concentration range 0.0050–0.2500 mg/mL, LoD = 0.0017 mg/mL, LoQ = 0.0050 mg/mL); and ferulic acid at 320 nm (y = 44,074.88x + 6.94, R^2^ = 1, 0.0014–0.0700 mg/mL, LoD = 0.0003 mg/mL, LoQ = 0.0008 mg/mL).

### 3.6. Antioxidant Capacity Testing

Antioxidant activity was assessed in vitro using free radical scavenging assays (DPPH and ABTS) and ferric reducing power assays (FRAP and TRC). The DPPH assay was conducted following Prieto’s microplate reader protocol (2012) [51], the ABTS assay according to Xiao et al. (2020) [52], the FRAP assay following the method of Benzie and Devaki (2018) [53], and the TRC assay based on the protocol of Mokrani et al. (2016) [54].

The results for the DPPH and ABTS assays are expressed as IC_50_ values, representing the concentration required to reduce free radical levels by 50%. For the FRAP and TRC assays, results are presented as milligrams of Fe^2^⁺ equivalents or milligrams of L-ascorbic acid equivalents per gram of dry weight, respectively. L-ascorbic acid and butylated hydroxytoluene, both well-known antioxidants, were used as control substances.

### 3.7. Enzyme Inhibitory Capacity

The *α*-amylase inhibition assay was conducted using the modified Caraway–Somogyi iodine-potassium iodide method as described by Zengin et al. (2014) [55]. A mixture of 25 µL of sample at varying concentrations and 50 µL of *α*-amylase (0.5 mg/mL in 6 mM NaCl in phosphate buffer, pH 6.8) was pre-incubated for 15 min at 37 °C in 96-well microtiter plates. Then, 0.2% starch solution was added, and incubation continued for another 20 min. The reaction was stopped by adding 25 µL of 1 M HCl, followed by the addition of 100 µL of I/KI solution for color development. Absorbance was measured at 630 nm using a Multiscan Sky ThermoScientific Plate Reader. Acarbose was used as the positive control.

The *α*-glucosidase inhibitory activity was assessed following the protocol of Wan et al. (2013) [56]. Various concentrations of the sample (120 µL) were pre-incubated with *α*-glucosidase (0.6 U/mL) for 15 min at 37 °C, followed by the addition of 3.5 mM *p*-nitrophenyl-*α*-D-glucopyranoside in phosphate buffer (pH 6.8). After a further 20 min of incubation, the reaction was stopped by adding 0.2 M sodium carbonate, and absorbance was measured at 405 nm. Acarbose was used as the positive control.

Cholinesterase inhibitory activity was assessed using Ellman’s reagent, following the method of Aktumsek et al. (2013) with slight modifications [57]. In 96-well microplates, 50 µL of sample at various concentrations was mixed with 25 µL of acetylcholinesterase or butyrylcholinesterase (0.026 U/mL) and 125 µL of DTNB (3 mM) in TRIS-HCl buffer solution (50 mM, pH 8). The mixture was incubated for 15 min at 37 °C. Then, 25 µL of acetyl/butyrylcholine iodide substrate was added, and after a further 20 min incubation at 37 °C, absorbance was measured at 405 nm. Galantamine was used as the positive control.

Tyrosinase inhibitory activity was evaluated according to Moonrungsee et al. (2012) [58]. A mixture of 50 µL of sample, 50 µL of mushroom tyrosinase enzyme, and L-DOPA substrate in 0.1 M phosphate buffer (pH 6.8) was incubated for 30 min at 25 °C. Absorbance was then measured at 475 nm, with kojic acid serving as the positive control.

The enzyme inhibitory activities were expressed as IC_50_ values, representing the concentration of extract or compound required to inhibit 50% of enzyme activity.

### 3.8. Antimicrobial Activity Testing

Antimicrobial activity was assessed using the broth microdilution method following CLSI guidelines (2015) [59]. Seven bacterial strains were tested, including Gram-positive strains (*Staphylococcus aureus* ATCC 6538, *Enterococcus faecalis* ATCC 29212, and *Bacillus subtilis* ATCC 6633) and Gram-negative strains (*Escherichia coli* ATCC 8739, *Klebsiella pneumoniae* NCIMB 9111, *Salmonella abony* NCTC 6017, and *Pseudomonas aeruginosa* ATCC 9027), along with one yeast strain (*Candida albicans* ATCC 10231). Overnight cultures of bacteria and yeast were grown in Mueller–Hinton broth and Sabouraud dextrose broth, respectively, at 37 °C. Cultures (100 µL), with concentrations of approximately 10^6^ CFU/mL for bacteria and 10^7^ CFU/mL for yeast, were transferred to 96-well microtiter plates. Tested extracts and compounds were first dissolved in a minimal volume of dimethyl sulfoxide (DMSO) and then diluted in the appropriate broth before being added to the microtiter plates (100 µL), ensuring a final DMSO concentration below 0.5%. Extracts were tested at concentrations of 62.5–1000 µg/mL, while compounds were assayed at 31.25–500 µg/mL. After 24 h of incubation for bacteria and 48 h of incubation for *C. albicans* at 37 °C, the minimum inhibitory concentration (MIC) was recorded as the lowest concentration showing visible inhibition of microbial growth. All tests were conducted in duplicate. Positive growth controls (200 µL), containing only bacterial or yeast cells in the medium (Mueller–Hinton broth or Sabouraud dextrose broth), were prepared for each microbial strain.

### 3.9. Statistical Analysis

Results are expressed as the mean ± standard deviation of the performed independent measurements. Data were analyzed using SPSS software (version 20, Chicago, IL, USA) with a one-way ANOVA followed by Tukey’s post hoc test or, where appropriate, an independent samples *t*-test. Statistical significance was defined as *p* < 0.05.

## 4. Conclusions

This study demonstrates that ethanol extracts from wheat and maize harvest residues exhibit weak to moderate bioactivity in in vitro antioxidant, enzyme-inhibitory, and antimicrobial assays. However, their ethyl acetate fractions and individual constituents display enhanced bioactivity, suggesting the potential of these agricultural by-products as sources of valuable compounds. However, their high lignocellulosic content presents a challenge, as active compound concentrations are relatively low. Therefore, to facilitate the practical application of these plant materials, developing simple and cost-effective methods to isolate or concentrate bioactive constituents is essential. These findings support further exploration into creating value-added products from wheat and maize residues, which could serve as raw materials in the pharmaceutical, cosmetic, and food industries.

## Figures and Tables

**Table 1:** Content of total phenolics, total flavonoids, and individual compounds in the wheat and maize harvest residues.

Parameter/Compound	Wheat	Maize
TPC EE (mg GAE/g) *	23.19 ± 0.00 ^a^	20.44 ± 0.23 ^b^
TPC EF (mg GAE/g)	27.830 ± 1.031 ^a^	12.407 ± 0.075 ^b^
TFC EE (mg QE/g) *	15.22 ± 0.00 ^a^	9.37 ± 0.83 ^b^
TFC EF (mg QE/g)	22.480 ± 1.994 ^a^	7.535 ± 0.092 ^b^
*p*-Coumaric acid (mg/g) *	2.140 ± 0.003 ^a^	1.641 ± 0.018 ^b^
Ferulic acid EE (mg/g) **	1.802 ± 0.010 ^a^	1.215 ± 0.027 ^b^
Tricin (mg/g) *	3.923 ± 0.001 ^a^	1.990 ± 0.016 ^b^
Salcolins A and B EE (mg/g) ***	4.373 ± 0.007 ^a^	6.192 ± 0.065 ^b^

Results are presented as the mean ± standard deviation (n = 3). Different superscript letters in the same row indicate statistically significant differences between values (*p* < 0.05). TPC—total phenolic content; EE—ethanol extract; EF—ethyl acetate fraction; GAE—gallic acid equivalents; TFC—total flavonoid content; QE—quercetin equivalents. * Concentration in wheat sample from publication of Lukić et al. (2024) [5] and in maize sample from publication of Glišić et al. (2024) [4]. ** Concentration of ferulic acid in maize sample from publication of Glišić et al. (2024) [4]. *** Calculated as tricin.

**Table 2:** Antioxidant activity of the ethanol extracts from wheat and maize harvest residues and their ethyl acetate fractions.

Assay	WEE	WEF	MEE *	MEF	BHT *	L-AA *
DPPH IC_50_ (mg/mL)	0.58 ± 0.01 ^b^	0.61 ± 0.01 ^a^	0.41 ± 0.02 ^c^	0.23 ± 0.02 ^d^	0.038 ± 0.001 ^e^	0.0057 ± 0.0003 ^f^
ABTS IC_50_ (mg/mL)	1.46 ± 0.04 ^b^	0.68 ± 0.01 ^c^	1.72 ± 0.10 ^a^	0.21 ± 0.01 ^d^	0.040 ± 0.002 ^e^	0.0316 ± 0.0003 ^e^
FRAP (µmol Fe^2+^/g)	0.23 ± 0.01 ^a^	0.32 ± 0.03 ^b^	0.25 ± 0.01 ^a^	0.62 ± 0.02 ^d^	0.41 ± 0.03 ^c^	1.618 ± 0.004 ^e^
TRC (mg L-AAE/g)	10.58 ± 0.66 ^a,b^	54.66 ± 1.27 ^b^	6.80 ± 0.05 ^a^	38.45 ± 2.80 ^a,b^	615.02 ± 38.87 ^c^	na

The results are based on three independent experiments performed in triplicate. Abbreviations: WEE—wheat ethanol extract; WEF—ethyl acetate fraction from wheat ethanol extract; MEE—maize ethanol extract; MEF—ethyl acetate fraction of maize ethanol extract; BHT—butylated hydroxytoluene; L-AA—L-ascorbic acid; L-AAE—L-ascorbic acid equivalents; na—not applicable. Different superscript letters within the same row indicate statistically significant differences between values (*p* < 0.05). * Data from Glišić et al. (2024) [4], except for TRC.

**Table 3:** Antioxidant activity of main compounds in ethanol extracts from wheat and maize harvest residues.

Assay	*p*-CA	FA	SAL-AB	TRN	BHT *	L-AA *
DPPH IC_50_ (mg/mL)	2.96 ± 0.16 ^a^	0.0068 ± 0.0001 ^c^	0.25 ± 0.01 ^c^	1.71 ± 0.15 ^b^	0.038 ± 0.001 ^c^	0.0057 ± 0.0003 ^c^
ABTS IC_50_ (mg/mL)	0.0256 ± 0.0012 ^c^	0.0034 ± 0.0001 ^d^	0.0310 ± 0.0011 ^b,c^	0.46 ± 0.01 ^a^	0.040 ± 0.002 ^b^	0.0316 ± 0.0003 ^b,c^
FRAP µmol Fe^2+^/g	0.92 ± 0.02 ^c^	2.02 ± 0.04 ^a^	0.95 ± 0.10 ^c^	0.34 ± 0.01 ^d^	0.41 ± 0.03 ^d^	1.618 ± 0.004 ^b^
TRC (mg L-AAE/g)	1810.89 ± 116.14 ^b^	2467.90 ± 91.26 ^a^	552.59 ± 12.44 ^c^	312.08 ± 12.44 ^d^	615.02 ± 38.87 ^c^	nt

The results are based on three independent experiments performed in triplicate. Abbreviations: *p*-CA—*p*-coumaric acid; FA—ferulic acid; SAL-AB—mixture of salcolins A and B (1:1); TRN—tricin; BHT—butylated hydroxytoluene; L-AA—L-ascorbic acid; L-AAE—L-ascorbic acid equivalents; nt—not tested. Different superscript letters within the same row indicate statistically significant differences between values (*p* < 0.05). * Data from Glišić et al. (2024) [4], except for TRC.

**Table 4:** Enzyme-inhibitory activity of the ethanol extracts from wheat and maize harvest residues and their ethyl acetate fractions.

Assay	WEE	WEF	MEE	MEF	ACB	GAL	KA
*α*-amilase IC_50_ (mg/mL)	90.07 ± 16.13 ^a^	7.31 ± 0.72 ^b^	>100	3.53 ± 0.65 ^b^	0.43 ± 0.02 ^b^	nt	nt
*α*-glucosidase IC_50_ (mg/mL)	19.55 ± 0.66 ^a^	10.23 ± 1.94 ^b^	5.80 ± 0.30 ^c^	0.11 ± 0.02 ^d^	0.37 ± 0.01 ^d^	nt	nt
AChE IC_50_ (mg/mL)	96.60 ± 10.56 ^a^	18.30 ± 0.07 ^c^	47.32 ± 6.59 ^b^	59.31 ± 2.08 ^b^	nt	9.66 ± 0.92 ^c^	nt
BChE IC_50_ (mg/mL)	76.37 ± 6.04 ^a^	56.16 ± 4.24 ^b^	>100	15.69 ± 1.63 ^c^	nt	9.87 ± 1.26 ^c^	nt
Tyr IC_50_ (mg/mL)	66.62 ± 4.25 ^a^	30.71 ± 3.97 ^b^	56.74 ± 6.96 ^a^	12.65 ± 1.62 ^c^	nt	nt	0.34 ± 0.07 ^d^

The results are based on at least two independent experiments performed in triplicate. Abbreviations: WEE—wheat ethanol extract; WEF—ethyl acetate fraction from wheat ethanol extract; MEE—maize ethanol extract; MEF—ethyl acetate fraction from maize ethanol extract; ACB—acarbose; GAL—galantamine; KA—kojic acid; AChE—acetylcholinesterase; BChE—butyrylcholinesterase; Tyr—tyrosinase; nt—not tested. Different superscript letters within the same row indicate statistically significant differences between values (*p* < 0.05).

**Table 5:** Enzyme-inhibitory activity of the main compounds in ethanol extracts from wheat and maize harvest residues.

Assay	*p*-CA	FA	SAL-AB	TRN	ACB	GAL	KA
*α*-amilase IC_50_ (mg/mL)	27.44 ± 2.38 ^a^	3.35 ± 0.85 ^b^	1.73 ± 0.16 ^b,c^	1.93 ± 0.23 ^b,c^	0.43 ± 0.02 ^c^	nt	nt
*α*-glucosidase IC_50_ (mg/mL)	1.10 ± 0.06 ^b^	1.49 ± 0.18 ^a^	0.44 ± 0.05 ^c^	0.19 ± 0.01 ^d^	0.37 ± 0.01 ^c,d^	nt	nt
AChE IC_50_ (mg/mL)	10.39 ± 0.20 ^a^	4.10 ± 0.37 ^c^	6.07 ± 0.43 ^b^	2.88 ± 0.06 ^c^	nt	9.66 ± 0.92 ^a^	nt
BChE IC_50_ (mg/mL)	19.81 ± 0. 47 ^a^	1.97 ± 0.08 ^c^	1.20 ± 0.27 ^c^	0.55 ± 0.02 ^c^	nt	9.87 ± 1.26 ^b^	nt
Tyr IC_50_ (mg/mL)	na	na	3.21 ± 0.72 ^a^	na	nt	nt	0.34 ± 0.07 ^b^

The results are based on at least two independent experiments performed in triplicate. Abbreviations: *p*-CA—*p*-coumaric acid; FA—ferulic acid; SAL-AB—mixture of salcolins A and B (1:1); TRN—tricin; ACB—acarbose; GAL—galantamine; KA—kojic acid; AChE—acetylcholinesterase; BChE—butyrylcholinesterase; Tyr—tyrosinase; na—not active in the tested concentration range; nt—not tested. Different superscript letters within the same row indicate statistically significant differences between values (*p* < 0.05).

**Table 6:** Antimicrobial activity of ethanol extracts from wheat and maize harvest residues and their individual compounds.

Microorganism	Minimal Inhibitory Concentrations MICs (μg/mL)					
	WEE	MEE	*p*-CA	FA	SAL-AB	TRN
Gram-positive bacteria						
*Staphylococcus aureus*	500	125	62.5	62.5	62.5	62.5
*Enterococcus faecalis*	250	125	31.25	125	31.25	31.25
*Bacillus subtilis*	125	125	31.25	62.5	31.25	31.25
Gram-negative bacteria						
*Escherichia coli*	>1000	>1000	>500	>500	>500	>500
*Klebsiella pneumoniae*	>1000	>1000	>500	>500	>500	>500
*Salmonella abony*	>1000	>1000	>500	>500	>500	>500
*Pseudomonas aeruginosa*	>1000	>1000	>500	>500	>500	>500
Yeast						
*Candida albicans*	500	>1000	250	>500	>500	250

Abbreviations: WEE—ethanol extract of wheat harvest residues; MEE—ethanol extract of maize harvest residues; *p*-CA—*p*-coumaric acid; FA—ferulic acid; SAL-AB—mixture of salcolins A and B (1:1); TRN—tricin.

## Data Availability

The raw data supporting the conclusions of this article will be made available by the authors upon request.

## Supplementary Materials

The following supporting information can be downloaded at: https://www.mdpi.com/article/10.3390/plants14030346/s1, Figure S1: Structure of tricin; Figure S2: Structure of salcolin A (tricin 4′-*O*-(*threo*-*β*-guaiacylglyceryl)ether) and salcolin B (tricin 4′-*O*-(*erythro*-*β*-guaiacylglyceryl)ether); Figure S3: Content (mg/g) of total phenolics, total flavonoids, and individual compounds in wheat ethanol extract (WEE) and maize ethanol extract (MEE).
