# Supplementary material for: Antioxidant, Enzyme-Inhibitory and Antimicrobial Activity of Underutilized Wheat and Maize Crop Residues
Source: Plants (Basel). 2025 Jan 24;14(3):346. doi: 10.3390/plants14030346 (PMC11820700; doi:10.3390/plants14030346)
Supplement: Supplementary file 1 [file plants-14-00346-s001.zip › plants-3366115-supplementary.pdf]

Supplementary Materials

Antioxidant, enzyme-inhibitory and antimicrobial activity of underutilized wheat and maize crop residues

Stevan Samardžić, Ivona Veličković, Marina T. Milenković, Jelena Arsenijević, Djordje Medarević, Zoran Maksimović

Correspondence: stevan.samardzic@pharmacy.bg.ac.rs; Tel.: +381-11-3951324

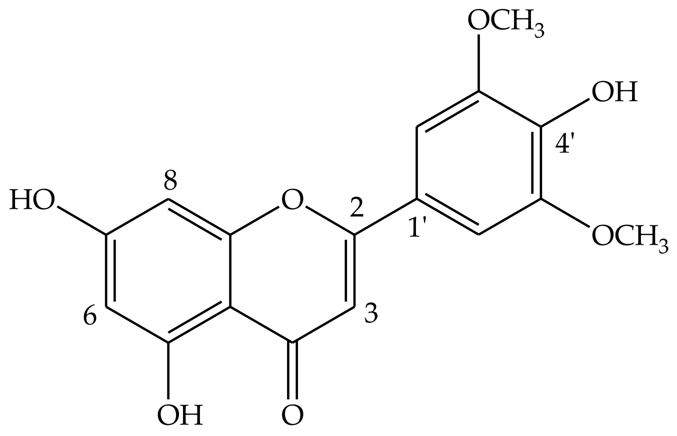

Figure S1. Structure of tricin.

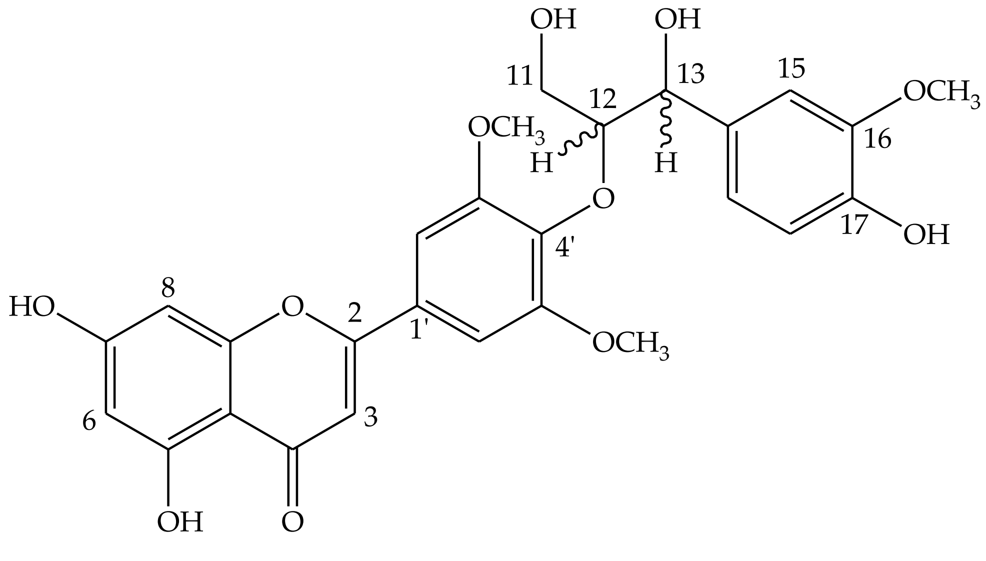

Figure S2. Structure of salcolin A (tricin 4′-O-(*threo*-*β*-guaiacylglyceryl)ether) and salcolin B (tricin 4′-*O*-(*erythro*-*β*-guaiacylglyceryl)ether).

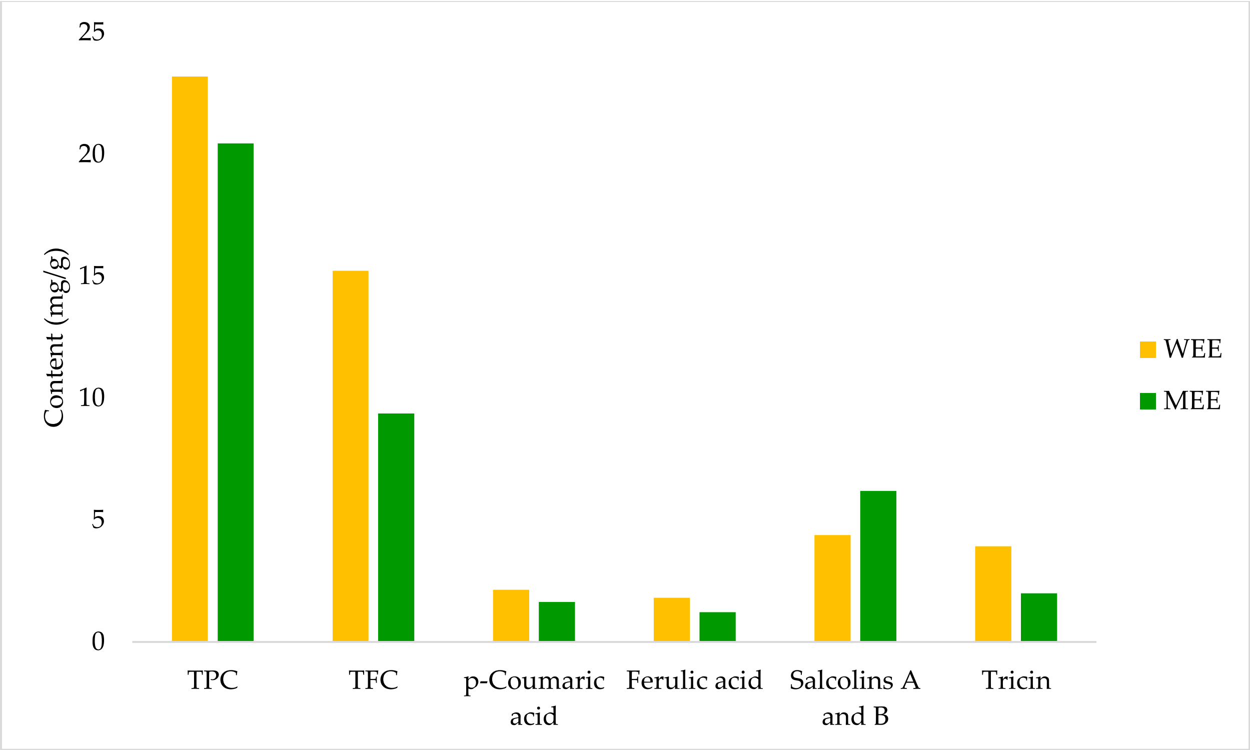

Figure S3. Content (mg/g) of total phenolics, total flavonoids and individual compounds in wheat ethanol extract (WEE) and maize ethanol extract (MEE). TPC – total phenolic content, TFC – total flavonoid content.
